# Which Milk during the Second Year of Life: A Personalized Choice for a Healthy Future?

**DOI:** 10.3390/nu13103412

**Published:** 2021-09-27

**Authors:** Elvira Verduci, Elisabetta Di Profio, Antonio Corsello, Lorenzo Scatigno, Giulia Fiore, Alessandra Bosetti, Gian Vincenzo Zuccotti

**Affiliations:** 1 Department of Health Sciences, University of Milan, 20146 Milan, Italy; 2 Department of Pediatrics, Vittore Buzzi Children’s Hospital, 20154 Milan, Italy; elisabetta.diprofio@unimi.it (E.D.P.); antonio.corsello@unimi.it (A.C.); lorenzo.scatigno@unimi.it (L.S.); giulia.fiore2@studenti.unimi.it (G.F.); alessandra.bosetti@asst-fbf-sacco.it (A.B.); gianvincenzo.zuccotti@unimi.it (G.V.Z.); 3 Department of Animal Sciences for Health, Animal Production and Food Safety, University of Milan, 20133 Milan, Italy; 4 Department of Biomedical and Clinical Sciences “L. Sacco”, University of Milan, 20157 Milan, Italy; 5 Pediatric Clinical Research Center Fondazione Romeo ed Enrica Invernizzi, University of Milan, 20157 Milan, Italy

**Keywords:** growing-up milk, milk formula, toddler, nutrients intakes, nutritional risks, iron deficiency, anemia, vitamin d deficiency, protein intake, second year of life

## Abstract

Nutrition in early life is a crucial element to provide all essential substrates for growth. Although this statement may appear obvious, several studies have shown how the intake of micro and macronutrients in toddlers differs a lot from the recommendations of scientific societies. Protein intake often exceeds the recommended amount, while the intake of iron and zinc is frequently insufficient, as well as Vitamin D. Nutritional errors in the first years of life can negatively impact the health of the child in the long term. To date, no clear evidence on which milk is suggested during the second year of life is yet to be established. In this study, we compare the nutrient profiles of cow’s milk and specific formulas as well as nutritional risks in toddlers linked to growth and childhood obesity development. The purpose of this review is to resume the latest clinical studies on toddlers fed with cow’s milk or young children formula (YCF), and the potential risks or benefits in the short and long term.

## 1. Nutritional Requirements during the Second Year of Life

When reaching the age of 12 months, children are usually referred to as toddlers. Indeed, the first 1000 days of life, the time from conception to the child’s second birthday, are considered a critical period for the healthy development of a newborn [1].

According to the Dietary Reference Values (DRV) of European Food Safety Authority [2], the energy requirements between 1 and 3 years of life vary according to sex and percentile of physical activity levels (PAL). On average, the energy requirements for a 1-year-old child with a 1.4 PAL is 790 kcal for males and 720 kcal for females, which increases to 1030 kcal and 960 kcal at 2 years and grows up to 1170 kcal and 1100 kcal at 3 years, respectively [2]. Early nutrition in toddlers implies a correct provision of macronutrient and micronutrient intakes to guarantee a healthy growth (Table 1).

Firstly, attention should be paid to the total protein intake during the beginning of life, when protein reference intake is estimated at 1.14–0.90 gr/kg/day for children aged 1–3 years [2].

Fat intake should not be limited before the 12 months of age, due to its importance in the neurologic development [3]. Between 1 and 3 years of age, fat recommended intakes are 35–40% of total daily energy intake (%En). Polyunsaturated fatty acids (PUFA) and monounsaturated fatty acids are the most recommended source of lipids. Among PUFA, particular attention is given to Linoleic Acid (LA) and Alpha-Linolenic Acid (ALA), whose recommended dietary intakes are 4%En and 0.5%En, respectively. On the other hand, saturated fats and trans-fats consumption should be limited as much as possible [2].

Long-chain PUFA, docosahexaenoic acid (DHA), and eicosapentaenoic acid (EPA) play an important role in brain development. It is known that the DHA status tends to decline during the complementary period [4], therefore toddlers’ diet should guarantee 250 mg daily of EPA and DHA, and for toddlers 1–2 years old a 100 mg daily increase of DHA should be provided [2].

Carbohydrates should provide the largest percentage of macronutrients, 45% to 60% of %En, in children 1–3 years old [2,3]. Carbohydrates such as vegetables, whole grains and beans are preferred over simple or refined, processed carbohydrates. In fact, the recommended amount of fiber daily intake for toddlers is 10 g. Added sugars should be avoided in children younger than 2 years and limited in older children, thus sugar consumption should be limited at less than 10%En [2].

The recommended daily intakes of minerals and vitamins for toddlers are summarized in Table 1. In toddlers there are some critical micronutrients, in which deficiencies have long-term consequences for growth and development as a child and may impact health as an adult. For example, data suggest that children aged 0–24 months may not be getting enough Vitamin D, iodine, or iron [1]. Vitamin D and calcium are essential for bone growth and bone mass acquisition during growth, the recommended daily intake of Vitamin D for children 1–3 years of age is 15 μg while the daily calcium intake is 450 mg [2]. Furthermore, among the preadolescent age groups, the highest prevalence of iron deficiency causing anemia has been found among toddlers [5]. Iron deficiency anemia in toddlers can be prevented by ensuring adequate iron intake during the first years of life, thus the daily recommended iron intake is 7 mg for toddlers [2].

## 2. Type of Cow’s Milks and Growing-Up Milk

The second year of life is a period of nutritional transition, and children achieve a mixed diet with a transition in their dietary pattern. After 12 months, in general, children should adapt to a diverse diet which includes fresh ingredients consumed together with the family [6]. Cow’s milk consumption is considered part of a balanced diet, and regular cow’s milk consumption should be avoided during the first year of life [7]. In fact, observational studies suggest that infants consuming large volumes of cow’s milk have a greater risk of iron deficiency and iron deficiency anemia due to lack of this critical micronutrient in cow’s milk [4]. During the second year of life, cow’s milk intake should be limited [7], because diets of infants and young children who consume a higher amount of cow’s milk have a significant difference in the macronutrient intakes if compared to breast-fed infants [4,7,8]. Like so, according to Early Nutrition Project Recommendations, reducing the dietary total protein content in infants’ diet could prevent an excessive early weight gain [7].

When consumed according to the appropriate nutritional guidelines, milk provides essential micro- and macronutrients to the diet. Cow’s milk is composed of about 87% of water, and it contains on average 3–4% of fats, 3.5% of proteins, about 5% of lactose and 1.2% of minerals. However, in the milk marketed for the direct consumption the fat content is usually standardized at three different levels (Table 2): whole cow’s milk (>3.5% fats), semi-skimmed milk (1.5–1.8% fats), and skimmed milk (<0.5%) [9].

Cow’s milk fat fraction is composed mainly of triacylglycerols, and about 60% of total fatty acids are saturated, above all palmitic acid (on average 30% of total fatty acids), followed by myristic and stearic acid. Other important constituents are unsaturated fatty acids, and oleic acid represents the main component (up to 18–20% of total fatty acids) [9,11]. Moreover, essential fatty acids linoleic and alpha-linolenic are present in cow’s milk, at about 1.5% and 1%, respectively, together with some cow’s milk-specific trans fatty acids, including conjugated linoleic acid (or CLA) [11].

Cow’s milk is a beverage rich in proteins of high biological value because they contain all essential amino acids and have a high digestibility and bioavailability. Finally, cow’s milk provides a variety of minerals, in particular calcium and phosphorus but also potassium, magnesium, zinc, selenium and both B-group water soluble vitamins and fat-soluble vitamins (such as Vitamins A and E), as listed in Table 2 [9].

Despite the cross-country variability of recommendations for milk, all international guidelines include cow’s milk consumption as a component of a balanced diet for children [9]. According to the Early Nutrition Project recommendations, the introduction of cow’s milk should be limited to no more than two cups a day in the second year of life [7]. In fact, regular cow’s milk includes about three to four times more protein per unit energy content than human milk or modern infant formula [7], bearing the risk of introducing higher protein intakes compared to recommended dietary allowance for toddlers [2].

Table 3 show the composition of whole cow’s milk, young child formula, and follow-on formula.

Italian guidelines for a healthy nutrition recommend daily consumption of 150 mL of cow’s milk among children 1–2 years, while between 2 and 3 years the daily portion is 200 mL [13]. Daily milk consumption provides digestible protein and calcium content essential for growth—however, milk has been placed at the bottom of the food pyramid defined by the Italian Society of Pediatrics [14], among the foods for which a daily consumption is suggested.

Interestingly, a recent systematic review and meta-analysis evaluated the relationship between regular cow-milk fat consumption (defined as daily or >4 times per week) and adiposity in healthy children ages 1 to 18 years. Whole fat milk consumption (3.25% fat) compared with reduced-fat (0.1–2%) milk, is associated with an adjusted OR of overweight or obesity of 0.61 (95% CI: 0.52–0.72; *p* < 0.0001), although heterogeneity was high [15]. As a result, whole fat cow’s milk consumption after 1 year has been associated with a lower risk of childhood overweight or obesity, compared with reduced fat cow’s milk. Between 1 and 2 years of life, there is no evidence to recommend a reduced-fat cow’s milk to prevent childhood overweight or obesity [16].

Increasing awareness of cow’s milk protein allergy and intolerance have influenced the market toward offering cow’s milk substitutes, which include milk from different mammalian species, special formula, and plant-based beverages. Being the former subject of analysis of two recent reviews, cow’s milk substitutes and their role will not be evaluated in this article [17,18].

Young child formulae (YCF), also known as toddler’s milk or growing-up milk (GUM), represent an alternative to cows’ milk or breast milk for children 1–3 years of age. YCF are milk-based, or plant-protein-based, formulae intended to partially satisfy nutritional requirements of young children, thus fortified with nutrients that are commonly low during the transition to family-based food (Table 2) [6,19]. There is not a legal definition for YCF nutrient composition, however a recommended nutritional composition has been proposed by the scientific community, considering critical nutrients and requirements of toddlers [20].

In view of the above, current diets offered to toddlers often do not meet proper daily nutrient requirements (Table 3). The European Safety Authority (EFSA) stated that dietary intakes of alpha-linolenic acid (ALA), docosahexaenoic acid (DHA), iron, Vitamin D, and iodine are low in infants and young children living in Europe and particular attention should be paid to ensuring an appropriate supply of these nutrients [12]. Conversely, dietary intakes of protein, salt, and potassium are generally high in young children across Europe [12].

According to the EFSA, fortified cow’s milk, fortified cereals, and cereal-based foods are efficient means to increase intakes of these critical nutrients [6,12]. However, in comparison with cow’s milk, currently marketed YCF contain more ALA, DHA (if added), iron and Vitamin D but similar amounts of iodine. Moreover, the mean content of these nutrients in young-child formulae is within the range of permitted concentrations in follow-on formulae in Europe (Table 2) [12,21].

YCF can be considered the best and most practical alternative to increase n−3 PUFA, iron, Vitamin D, and iodine intakes in toddlers [6], although no unique role in the provision of critical nutrients has been given to them when compared to other foods that may be included in the normal diet of young children (such as follow-on formulae and cow’s milk) [12].

Furthermore, there is no international legal definition or compositional criteria for these products, leading consequently to a different spread of products all over European countries. The EFSA panel up to date have not considered the necessity to propose specific compositional criteria for YCF, as the formulae is consumed during the first year of life and continues to be used by young children [21]. Moreover, the European Society for Paediatric Gastroenterology, Hepatology and Nutrition (ESPGHAN) states there is no need of a daily routine use of YCF among children aged 1–3 years [6]. However, the lack of compositional guidelines is a matter of concern, and some recent studies provided recommendations for YCF composition [20,22] to avoid an inappropriate composition and ensure adequate intake for the target population.

Recently, the International Expert Group coordinated by the Early Nutrition Academy has recommended daily servings of 1 to 2 cups (200–400 mL) of YCF, which provide approximately 15% of total energy intake in young children [20].

Lastly, the authors advocate the need of clearly divided marketing of YCF, from those intended for infant to the follow-on formula [6,23]. It is important that parents understand the differences among those products, and why YCF contributes less to the nutrient intake of a toddler than an infant formula does for the infant [6].

## 3. Nutritional Risks in Toddlers

### 3.1. Growth, Obesity, and Protein Leverage Hypothesis

Obesity represents an increasingly current and frequent problem during the pediatric age. A study conducted in Canada showed that the 28.4% of children between 5 and 19 years can be considered overweight or obese. However, even in the age group between 0 and 5 years, the prevalence observed is quite high, approximately 6% [24]. Despite this evidence, there is not still a definition of excessive adiposity in children under 2 years of age. Moreover, World Health Organization (WHO) suggests evaluating children by comparing them using growth curves, such as weight-for-length percentiles (WFL) or Body Mass Index (BMI), allowing to define them overweight or obese according to specific percentiles cut-offs [25].

A study published in 2016 by Roy et al. involving 73,949 infants tried to compare two different methods of measurement, in order to define which could better predict a future risk of developing obesity [26]. It emerged that an alteration of the BMI in early childhood was more associated to the risk of developing obesity when compared to an alteration of the WFL. However, after the sixth month, a comparison between BMI and WFL curves was comparable. Therefore, the recommendation that emerged from these findings was to always combine the measurement of WFL with the measurement of BMI in the first years of life, to better identify children at higher risk of developing obesity, and, subsequently, trying to prevent it through an early dietary intervention.

Diet indeed represents one of the most critical factors for physiological growth, particularly in early stages, with the introduction of complementary feeding. Further evidence suggests that this period could influence not only the initial stages of life but could also have an impact on the pathogenesis of obesity and subsequent diseases even in adolescence and adult life [27]. In this regard, recent studies found that a rapid weight gain in the early stages of life is closely linked to an increased risk of developing overweight and obesity in childhood and adolescence, with higher risk of developing correlated non communicable diseases, such as type 2 diabetes and arterial hypertension [28,29]. Other frequent causes to these conditions in young children are socio-cultural factors, genetic and dietary habits, such as increased protein consumption in infants or excessive fat intake in older ages.

Furthermore, although the WHO recommends continuing breastfeeding in children up to 2 years of age, this event has been observed to be rarely fulfilled among the general population [30]. Breastfeeding rates decrease rapidly with increasing age and only a few infants are breastfed until one year of life, and cow’s milk is frequently introduced into the diet replacing breast milk [31]. Cow’s milk contains approximately 3 times as much protein as human milk. Moreover, reduced fat (skimmed) milk even doubles the protein contained in whole cow’s milk [32]. Even most of the formula milks, based on cow’s milk, contain a higher amount of protein than human milk, although much less if compared to commercial cow’s ones [33].

The result of this milk-based nutrition with an increased concentration of protein, along with other “feeding errors”, means that children in late infancy and toddlers are usually provided with a protein intake that is 3 or 4 times higher than the physiological requirements necessary to ensure a proper growth, even with a wide variability observable.

As numerous studies have shown, a diet rich in total amount of proteins, especially during the first years of life, correlates with a higher risk of developing overweight and obesity in childhood and adolescence. The theory behind this evidence is known as the “early protein hypothesis” [34]. The DONALD study has shown that children who are fed a high-protein diet in the first 18–24 months present an increased risk of developing overweight and obesity by the age of 7 [35]. As a further confirmation of this theory, a very similar fact has also been reconfirmed by another recent study, where a strong association between the total amount of proteins supplied in the first year of age, particularly those coming from dairy products, and becoming overweight at 5 years of age [36].

The pathophysiological mechanism underlying this observation seems to be related to the increase in proteins introduced with the diet, which determines an increase in amino acids in the bloodstream and a subsequent greater secretion of insulin and insulin-like-growth factor-1 (IGF-1), which is able to activate the mTOR growth signaling network [37]. The increase of these circulating anabolic hormones and factors could therefore increase the deposition of lipids in the adipose tissue, contributing significantly to the increase in weight, BMI and WFL observed in children fed with cow’s milk-based formulas or commercial cow’s milk [38].

An important confirmation of the early protein hypothesis came from the EU Childhood Obesity Project (CHOP) [39]. In this study, 1670 children born in the European Union countries were enrolled, and two groups were created. The first group was fed with formulas that contained the minimum amount of protein recommended, while the second one was fed with formulas that contained the maximum recommended. From this randomized trial it emerged that children in the low protein group showed lower weight than the high protein group at 2 years, with a comparable length average. Moreover, among the same children observed in the follow-up, overweight and obesity at 6 years were significantly more present in the group that had received high-protein formulas than in the low-protein one (10% and 5%, respectively, in terms of obesity prevalence rate).

Furthermore, cow’s milk contains two major protein fractions, which are known as casein (80%) and whey (20%) proteins. Differences in digestibility between whey and casein protein have been found in both adults and infants, with a more rapid digestibility of whey proteins versus casein ones and a subsequent relatively lower digestion efficiency [40]. Further evidence showed that differences between undigested peptides ratio could also be involved and have an active role on the colonic microbiota composition, even with limited findings in humans at the moment [41]. Indeed, possible antimicrobial action of enzymatic digestion products of both whey and casein proteins have been found in vitro, with subsequent growth stimulatory effects on beneficial bacteria, such as *Bifidobacteria* [42].

These data therefore seem to suggest that an increase in protein in infancy could correlate with an increased risk of weight gain or possible intestinal dysbiosis. This evidence seems to be true when it comes to dairy and milk proteins and formulas, but when other animal and non-animal protein sources enter the equation, the situation becomes more complex [43]. As an example, recent studies seem to suggest that the introduction of meat proteins into the diet is associated with physiological and linear growth in length without a real risk of weight gain and pathological increase in BMI [34]. Nowadays, therefore, many questions remain open, and there are still few randomized and controlled studies that have dealt with this issue. The clear role of proteins from different sources on a child’s growth partially remains uncertain, and further research is needed on this topic.

Another relevant evidence about dietary influence of toddlers’ diet on growth emerges in the review by Agostoni et al. about the role of fats in the first two years of age [44]. They found that lipid intake in the first 24 months was not associated in any way with overweight and obesity in later ages, and this data was further confirmed by Rolland et al., who even found a negative correlation between the hyperlipidic diet of a toddler and sera leptin levels at 20 years, the main anorexigenic hormone capable of being produced by the adipose tissue and the fat mass [45]. For these reasons, a diet with a total lipid amount exceeding the recommended 35–40% of the total energy daily intake during the first months of life could even be protective against the development of overweight and obesity in childhood and adolescence [33].

Furthermore, understanding the underlying mechanisms of exclusive breastfeeding prevention over a later obesity could strength the importance of breastfeeding, and it might also help to improve formulas, complementary foods, and dietary habits for the toddler [38,46].

### 3.2. Micronutrient Deficiencies

Another common nutritional problem of infants and toddlers can be represented by the lack of micronutrients [1]. Most common causes are generally caused by problems with breastfeeding, errors in the preparation and administration of formulas and supplementation, and by the child’s refusal of food, also known as “picky eating”, a frequent phenomenon in toddlers, with an estimated prevalence reaching 25–32% [47].

Another relevant criticality may be linked to the fact that, around the age of 6 months, infant’s reserves of micronutrients such as zinc and iron are almost completely depleted, and the metabolic demand of the same micronutrients increases significantly due to rapid growth in length and weight. For this reason, it is fundamental that feeding in the first 2 years of life follows this physiological increase in demand, in order to avoid deficiency episodes, both in the short and long term, particularly among high-risk patients, such as low birth weight newborns [48].

Micronutrients, such as iron and zinc, are fundamental for the neurological and stature development of the child, while Vitamins D, B12, and B6, and folate are fundamental to improve bone health and avoid deficiencies that could cause diseases such as anemia or rickets. These deficiency episodes can be very common in young children: as an example, exclusively breastfed preterm newborns can be on a higher risk of incurring in zinc deficiency, with subsequent possible alopecia, diarrhea, dermatitis and, in severe forms, even cognitive development delays and immune-related disorders [49,50].

Iron deficiency is one the most relevant and common deficiencies during the first 2 years of life, and it has been found in about the 8% of children between 12 and 24 months, in which 4% suffer from iron-related anemia [51,52]. As for zinc deficiency, most at risk patients are preterm infants, followed by children who consume high quantities of cow’s milk [53]. The consumption of cow’s milk in quantities greater than 450 mL/day has been shown to be associated with a higher risk of iron deficiency anemia [54]. For this reason, the ESPGHAN suggests that children should receive foods containing a high amount of iron and that the consumption of cow’s milk in quantities exceeding 500 mL/day be avoided [5]. Moreover, the awareness of possible iron deficiencies, even in toddlers, is always an important element in the clinical practice, and it can be easily avoided with a simple and accurate nutritional intervention [52].

Finally, other relevant micronutrients that are frequently deficient in young children are docosahexaenoic acid (DHA) and Vitamin D. It has been shown that almost all children in the United States have a DHA daily intake lower than the recommended one, while, on the other hand, it is proven that the correction of this deficit is correlated with an improvement in the respiratory capacity [55,56]. It is also known that DHA has an important neuroprotective role and favors the cognitive development of the child [57].

Vitamin D deficiency even represents a more concerning issue: recent reports conducted on US children has shown that about 50% of children under 24 months have a Vitamin D deficiency, and more than 70% of them do not meet the adequate intake that is recommended [1]. Even if no association was found between sera Vitamin D levels and body length-for-age and head circumference in the first years of life, long term effects of a misunderstood severe deficiency in a toddler could be various and even irreversible, also including extra-skeletal problems, such atopy and autoimmunity disorders [58].

## 4. Which Milk Is the Best Choice during the Second Year of life? Evidence from Clinical Trials

Nutritional adequacy of diet in children between 12 to 36 months is still a challenge regarding several nutrients as mentioned before. In the last years, GUM has been investigated regarding its safety and adequacy in order to reach dietary reference intakes, being considered both by defect and excess when compared to whole cow’s milk.

In 2013, a randomized controlled trial [56] was conducted on 85 Irish children aged 12–24 months divided in two groups, with a cow’s milk only consumption group (*n* = 56) compared to a GUM (≥100 g/day) plus cow’s milk consumption group (*n* = 29). Children had an average daily total milk intake of about 300 g. A four-day weighed food diary was used to collect detailed food and beverage intake data. They found a similar Mean Daily Intake (MDI) in both groups for energy, total fat, sodium, calcium, thiamine, riboflavin, niacin, folate, and Vitamin A. The GUM group presented a significantly lower intake of protein, saturated fat, and Vitamins B6 and B12, while a higher intake of carbohydrates, dietary fiber, iron, zinc, and Vitamins C and D. In both groups the mean protein intake was 3.4–3.6 g/kg body weight per day. Mean dietary fiber and total iron intake were found to be higher in the GUM group (93%), while in cow’s milk group 59% of children presented intakes inferior to the Estimated Average Requirements (EAR). Moreover, Vitamin D intakes were below UK Recommended intakes in both groups. Authors [59] concluded that GUM could contribute to reach proper amounts of energy, macro- and micronutrients.

In 2018 a multicenter, double -blind, randomized controlled trial [60] was conducted in New Zealand on 160 healthy children aged 1 year ± 2 weeks. The intervention group required the consumption of 300 mL/day of a supplied lite milk (GUMLi) fortified with iron, cholecalciferol, probiotics, prebiotics but reduced in protein, for one year. The control group consumed non fortified Cow’s Milk (CM). Sixty-seven children for each group completed the study. The primary outcome was to detect the effect on body fat percentage at 2 years of age after daily consumption of an energy and protein-reduced GUM. Results showed that the estimated mean difference in percentage body fat between the intervention and control at 6 months was −0.46% (95% CI: −2.54, 1.61; *p* = 0.66) and at 12 months was −2.19% (95% CI: −4.24, −0.15, *p* = 0.036). They used a validated age and gender-appropriate equation and, to detect Fat Free Mass, a constant age- and gender-specific hydration was used. No significant differences were found in weight, BMI, zBMI, and weight-length z-score at 6 months and 12 months of intervention. The mean of daily energy intake were similar between two groups, however, protein intake was significant lower in the intervention group (46 ± 10 g/day vs. 51 ± 12 g/day). Authors concluded that children who had an inferior dietary protein intake presented a body fat mass of 1.58% lower if compared to the cow’s milk group, who consumed higher protein amounts. Moreover, authors declare the importance of proper complementary feeding in a child’s life. Indeed, an excessive protein intake that overcomes the recommendations is frequently observed, caused by a progressive substitution of breast milk or formula by solid food.

The early protein hypothesis, which link the amount of daily protein and childhood obesity is nowadays well known, and animal proteins, including milk proteins, have been positively associated to the increase in serum insulin-like growth factor concentrations in 2.5-year old children [61]. From the same court [62], dietary iron and vitamin D intakes and prevalence of iron deficiency (ID), iron deficiency with anemia (IDA) and vitamin D deficiency (VDD) were investigated. Dietary intake was detected using Eating Assessment in Toddlers Food Frequency Questionnaire (EAT FFQ) at 3, 6, 9, and 12 months of study. Results showed a lower development of ID after 12 months in the GUMLi group (7%) if compared to the CM group (24%). Also, an improvement in Vitamin D status was reported in GUMLi group with a decrease in VDD to 3%, whereas the CM group results showed an increase of 14%. Furthermore, 25(OH)D levels after 12 months of fortified milk consumption, measured during the winter months, showed an average of values in the normal range, highlighting how this milk is useful for maintaining 25(OH)D concentration to prevent VDD.

In a subgroup of the GUMLi Trial (*n* = 83 children) the Probability of Adequate Nutrient Intake (PANDiet) was used to analyze the nutritional adequacy of diets of participants [63]. A 24 h recall diary in both groups was performed. In the CM group, riboflavin and potassium were higher whereas in GUMLi group Vitamin C and iron where higher. Some nutrients were below reference value for both groups; Vitamin D, copper, and iodine. Consuming GUM was associated with higher nutritional adequacy of diets in children aged 18 to 23 months. GUMLi consumers had carbohydrate and Saturated Fatty Acid (SFA) intake in line with recommendations, but still presented an excessive protein intake.

In the GUMLi study, authors validated the Eating Assessment in Toddlers FFQ (EAT FFQ) [64], that estimate the content of protein, total fat, carbohydrate, fiber, Ca, Fe, Zn, and Vitamins B12, C, and D. Subsequently they administered the EAT FFQ [19], which describe dietary intake over previous 4 weeks at 3, 6, 9, and 12 months of the intervention.

They found that GUMLi improved the intakes of iron, Vitamins D and C, Zinc and reduced protein intakes if compared to unfortified CM. EAT FFQ was able to describe dietary patterns and differences both for food and nutrient intakes in toddlers. Indeed, they identified three dietary patterns: “junk/snack pattern”, “healthy/guideline pattern”, “breast milk/formula pattern”. The first two were similar and positively associated to several nutrients’ intake, and the “healthy/guideline pattern” reflected a more nutrient profile. On the contrary, the “breast milk/formula pattern” had a negative association to most of the nutrients detected.

Authors concluded that the studies conducted had focused on a single-nutrient intake [65,66,67], but on the other hand, using EAT FFQ it is possible to describe multiple-nutrient intakes, showing how GUMLi consumption has less protein intake and higher intake in key nutrients (i.e., iron and Vitamin D).

Lastly, a recent published paper [68] investigated the effect of reduced-protein GUMLi vs. CM on protein intake, growth, and plasma IGF-1 at 2 years old. A subgroup of 79 children participating in GUMLi study were involved. They found no differences in length-for-age and weight-for-age z-score between two groups but a lower mean body fat % of 3.2% in the GUMLi group at 2 years. Moreover, after 12 months of intervention, a significant association between IGF-1 at 2 years of life and total cow’s milk intake was found. They also performed a hierarchical multiple linear regression incorporating sex and length-for-age to check whether it was independent or confounding and the association was still significant. This evidence suggested the importance of the influence of CM on IGF-1 concentrations and growth after 1 year of life but do not allow inferring causality or determining the increased risk associated with exceeding the current protein recommendations in the second year of life due to insufficient numbers in each group to detect clinically significant differences in outcomes [68].

Indeed, these studies were conducted on toddlers in New Zealand, which might present food sources (i.e., fruits or vegetables) or habits (i.e., breakfast composition) different from European toddlers’ population, with a distinct genetic susceptibility.

## 5. Conclusions

Meeting nutritional requirements in toddlers is often a challenge, especially for iron, zinc, Vitamin D, EPA, and DHA. Cow’s milk is routinely included in children’s diet from 12 to 36 months. The period in which children’s diets become diversified due to the introduction of solid foods is crucial, and an excessive protein intake (3 or 4 times/kg/day than the EFSA Dietary Reference Values [2]) has been observed in this population, with related deficiencies of iron, Vitamin D, and other micronutrients such as zinc [49,50].

National Italian guidelines for a healthy nutrition recommend a daily consumption of 150 mL of cow’s milk among children aged 1–2 years, while between 2 and 3 years the daily portion should correspond to 200 mL [13]. However, international recommendations [7] suggest not to consume more than 2 cups a day of cow’s milk, so as to not exceed the daily protein requirement. Moreover, toddlers consuming cow’s milk are more at risk of developing iron or Vitamin D deficiencies [4]. Instead, the International Expert Group coordinated by the Early Nutrition Academy recommended YCF consumption at doses of 1–2 cups (200–400 mL) daily, which provide approximately 15% of the total energy intake in young children [20] fortified with all critical micronutrients for toddlers.

Most recent ESPGHAN recommendations do not suggest mandatory use of YCF [6]. A European Regulation is still lacking for a YCF nutrient composition definition; nevertheless, a recommended nutritional composition has been proposed by the scientific community, considering critical nutrients and requirements of toddlers [20].

Randomized clinical trials have been conducted in the last years regarding YCF consumption. An early development of overweight and obesity and higher concentrations of IGF-1 at 2.5 years of age have been associated with an excessive amount of protein intake [35], and some studies [19,60,62,64] have shown that children who consumed YCF had a lower protein intake than those who consumed cow’s milk. Moreover, results showed that toddlers who consumed YCF with a lower protein content than cow’s milk had a lower percentage of body fat mass [60,62], and that IGF-1 concentration at 2 years was positively associated with the total cow’s milk intake [68].

**Figure 1:**
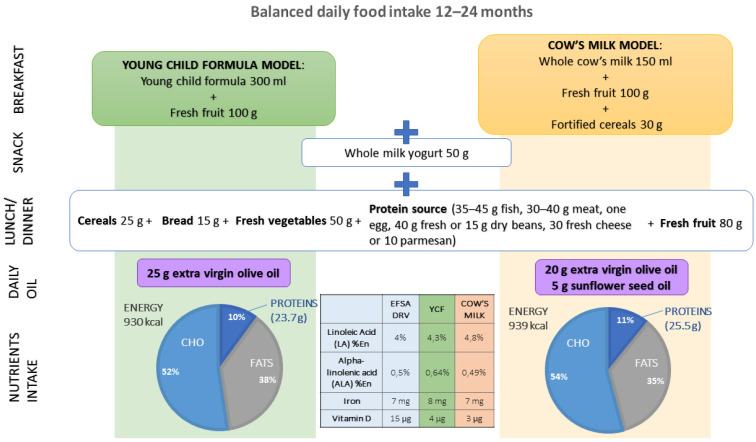
Suggested daily food intake for 12–24-month-old children depending on the type of milk consumed (young child formula, cow’s milk) to ensure a nutrients intake within the range of reference values.

Thus, novel approaches in managing toddler’s nutrition should underline which type of milk is a better choice according to individual needs. When consuming a YCF alongside a balanced diet, nutritional requirements are more easily ensured, thanks to formulas specifically developed for toddlers. By contrast, dietary patterns including cow’s milk should address daily consumption of fortified cereals and sunflower oil, together with olive oil to obtain an optimal macro- and micro-nutrients intake (Figure 1).

In conclusion, personalized dietary strategies could be favorable in promoting a healthy diet with an optimization of nutrients’ intake. However, future studies are needed to better underline the role of YCF on growth and health of toddlers, and their possible effects in the long term.

## Figures and Tables

**Table 1:** Daily nutrient requirements and reference intakes in toddlers (1–3 years) ^1^.

Proteins	
Pro kg	1.14–0.90 g/kg a day
Fats	35–40% %En
Saturated fatty acids	As low as possible
Linoleic Acid (LA)	4%
Alpha-Linolenic Acid (ALA)	0.5%
EPA + DHA	250 mg + 100 mg (during the second year of life)
Trans fatty acids	As low as possible
Carbohydrates	45–60% %En
Sugars	<10%
Fiber	10 g
Minerals	
Sodium (g)	1.1
Potassium (g)	0.8
Chloride (g)	1.7
Calcium (mg)	450
Phosphorous (mg)	250
Magnesium (mg)	170–230
Iron (mg)	7
Zinc (mg)	4.3
Copper (mg)	0.7–1
Manganese (mg)	0.5
Fluoride (mg)	0.6
Iodine (μg)	90
Selenium (μg)	15
Vitamins	
Vitamin A (μg)	250
Vitamin D (μg)	15
Vitamin E (mg)	6–9
Vitamin K (μg)	12
Vitamin B1 or thiamine (mg)	0.1
Vitamin B2 or riboflavin (mg)	0.6
Vitamin B3 or niacin (mg)	1.6
Vitamin B5 or pantothenic acid (mg)	4
Vitamin B6 or pyridoxine (mg)	0.6
Vitamin B7 or biotin (μg)	20
Vitamin B9 or folic acid (μg)	120
Vitamin B12 or cobalamin (μg)	1.5
Vitamin C (mg)	20

^1^ Dietary Reference Values (DRV) in toddlers [2]. %En = total daily energy intake, DHA = docosahexaenoic acid, EPA = eicosapentaenoic acid.

**Table 2:** Nutrient content in 100 g of different type of commercially available cow’s milks ^1^.

	Whole Cow’s Milk	Partially Skimmed Milk	Skimmed Milk
Energy, kcal	64	46	36
Water, g	87	88.5	90.5
Carbohydrate, g	4.9	5.0	5.3
Protein, g	3.3	3.5	3.6
Fat, g	3.6	1.5	0.2
Polyunsaturated Fatty acids (PUFA), g	0.12	0.08	0.01
Linoleic acid, g	0.07	0.05	0.01
Linolenic acid, g	0.05	0.03	traces
Eicosapentaenoic acid (EPA), g	0.00	0.00	0.00
Docosahexaenoic acid (DHA), g	0.00	0.00	0.00
Calcium, mg	119	120	125
Sodium, mg	50	46	52
Phosphorus, mg	93	94	97
Iron, mg	0.1	0.1	0.1
Zinc, mg	0.38	0.37	0.59
Potassium, mg	150	170	150
Magnesium, mg	12	11	11
Selenium, µg	1.6	1.6	1
Vitamin D, µg	0.03	0.01	traces
Vitamin A, µg (RE-eq)	43	21	traces
Vitamin E, mg	0.07	0.04	traces
Vitamin C, mg	1	1	1

^1^ Data from [10].

**Table 3:** Nutrient composition of cow’s milk and young infant formula on average compared to EFSA recommendation for follow-on formulae ^1^.

	Whole Cow’s Milk (Mean)	Young Child Formula, (Median)	EFSA Recommendation for Follow-On Formula (Min-Max or Min)
Energy (kcal/100 g)	69	67	60–70
Proteins (g/100 kcal)	4.8	2.6	1.6–2.5
Casein		1.7	
Whey protein		0.7	
Fats (g/100 kcal)	6.1	4.3	4.4–6
Saturated fat (g/100 kcal)		1.4	
Monounsaturated (g/100 kcal)		1.9	
Polyunsaturated (g/100 kcal)		0.9	
Linoleic acid (g/100 kcal)	0.07	0.8	0.5–1.2
Arachidonic acid (g/100 kcal)		0	
Alpha-linolenic acid n−3 (mg/100 kcal)		103	50–100
Eicosapentaenoic acid (EPA) (mg/100 kcal)		19	
Docosahexaenoic acid (DHA) (mg/100 kcal)		6.4	20–50
Trans fatty acids		6.4	<3% of total fatty acids
Carbohydrates (g/100 kcal)	6.8	12.6	9–14
Total sugars (g/100 kcal)		9.9	<20% of total CHO
Lactose (g/100 kcal)		9	>4.5
Sucrose (g/100 kcal)		2.1	
Glucose (g/100 kcal)		0.5	0
Maltose (g/100 kcal)		0.2	
Maltodextrin (g/100 kcal)		4.1	
Fiber		0.8	
Minerals			
Sodium (mg)	64.3	40.4	25
Potassium (mg)	215.1	126.8	80
Chloride (mg)	146.5	75	60
Calcium (mg)	176.7	126.9	50
Phosphorous (mg)	138.3	77.6	25
Magnesium (mg)	16.8	10.4	5
Iron (mg)	<0.1	1.8	0.6
Zinc (mg)	0.6	1.1	0.5
Copper (mg)	0	0.1	0.06
Manganese (mg)	0	0	1
Fluoride (mg)		0	not necessary
Iodine (μg)	23	20	15
Selenium (μg)	1.9	2.4	3
Chromium (μg)		1.4	not necessary
Molybdenum (μg)		4.2	0.4
Vitamins			
Vitamin A (μg)	57.5	101.6	70
Vitamin D (μg)	0.1	2.1	2
Vitamin E (mg)	0.1	1.6	0.6
Vitamin K (μg)	0	7.5	1
Vitamin B1 or thiamine (μg)	0	0.1	0.04
Vitamin B2 or riboflavin (μg)	0.3	0.2	0.06
Vitamin B3 or niacin (mg)	1.0	0.9	0.4
Vitamin B5 or pantothenic acid (mg)	0	0.7	0.4
Vitamin B6 or pyridoxine (μg)	0	0.1	0.02
Vitamin B7 or biotin (μg)	4.3	3.1	1
Vitamin B9 or folic acid (μg)	9.1	22.4	15
Vitamin B12 or cobalamin (μg)	0.7	0.3	0.1
Vitamin C (mg)	1.9	15.4	4

^1^ Modified from [6,12].

## Data Availability

Not applicable.
